# Perioperative Nicotine for Postoperative Pain and Nausea: A Systematic Review with Clinical and Methodological Insights

**DOI:** 10.5812/aapm-164878

**Published:** 2025-11-21

**Authors:** Feras Twfeq Almogbel, Mohammed Ali Alghamdi, Ali Mohamed Elkarouri, Abdulrahman Fahad Alharbi, Layan Saud Almutairy, Lama Zaid Alzimami, Atheer Alkhalil Medkhali, Reem Abduldaem Aloufi, Shatha Muways Alotaibi, Rawan Fauzy Allehyani, Jawaher Hani Alabdualqader, Mona Jalal Awaji

**Affiliations:** 1 Dammam Medical Complex, Nephrology Clinical Pharmacy Consultant, Dammam, Saudi Arabia; 2 Department of Pharmaceutical Affairs, Dammam Medical Complex, Dammam, Saudi Arabia; 3 College of Medicine, University of Medical Sciences and Technology, Khartoum, Sudan; 4 Department of Pharmaceutical Care, King Faisal Specialist Hospital and Research Centre, Madinah, Saudi Arabia; 5 Department of Pharmacology, Batterjee Medical College, Jeddah, Saudi Arabia; 6 College of Medicine, King Faisal University, Al Ahsa, Saudi Arabia; 7 Department of Pharmacology, Al Nahdi Medical Company, Jizan, Saudi Arabia; 8 Department of Pharmacology, Taibah University, Madinah, Saudi Arabia; 9 Department of Pharmacology, Umm AL-Qura University, Makkah, Saudi Arabia; 10 Department of Pharmacology, Umm AL-Qura University, Makkah, Saudi Arabia; 11 Department of Pharmacy, King Faisal University, Al Ahsa, Saudi Arabia; 12 Jazan Health Care Cluster, Nursing Department, Jazan, Saudi Arabia

**Keywords:** Nicotine, Postoperative Nausea and Vomiting (PONV), Opioid, Pain Management

## Abstract

**Context:**

Nicotine has been investigated in prior studies for its analgesic effects and its impact on postoperative nausea and vomiting (PONV), yet results have been inconsistent.

**Objectives:**

This systematic review and narrative synthesis evaluates the effects of perioperative nicotine administration on postoperative pain control and PONV in patients undergoing general anesthesia.

**Methods:**

A systematic literature review was conducted, and findings were summarized narratively. Comprehensive searches were performed in PubMed, the Cochrane Central Register of Controlled Trials (CENTRAL), and Google Scholar for studies published between 2004 and 2023, using a PICO-based approach. The PICO criteria included: Patients undergoing general anesthesia, perioperative nicotine as the intervention, placebo or no nicotine as the comparator, and pain scores as the primary outcome. The Mendeley application was utilized to eliminate duplicate data. Title, abstract, and full-text screenings were independently conducted by all authors using the online review platform Rayyan. Final data were individually extracted into Excel spreadsheets. The risk of bias in the included studies was assessed with the Cochrane Risk of Bias 2 (RoB 2) tool.

**Results:**

Eleven studies encompassing 753 participants (384 receiving nicotine, 369 controls) were included. Of these, 514 were female and 239 were male, all having undergone different surgical procedures and receiving nicotine via various methods and dosage forms. The majority of participants were nonsmokers. Primary outcomes across the studies predominantly involved postoperative pain scores, while secondary outcomes included the incidence of PONV, antiemetic requirements, and opioid consumption. No additional analyses were performed due to heterogeneity among the included studies.

**Conclusions:**

Although perioperative nicotine administration demonstrated reductions in postoperative pain, nausea, vomiting, and opioid consumption in some studies, the effect of nicotine on PONV was inconsistent. Variability in patient populations, dosage forms, and dosages complicates the formulation of definitive clinical recommendations. Overall, perioperative nicotine shows promise for improving postoperative pain management, but its impact on PONV requires careful consideration. Nicotine administration has been investigated as an analgesic adjunct and as a strategy for preventing PONV. This systematic review aimed to determine the effect of perioperative nicotine administration on postoperative pain and PONV.

## 1. Context

Postoperative pain, nausea, and vomiting remain significant clinical challenges for healthcare providers. Inadequate management of these symptoms can delay recovery, reduce patient satisfaction, prolong hospitalization, and increase healthcare costs (1, 2). Despite advances in pharmacological management, 70 - 80% of surgical patients in the United States still experience moderate to severe postoperative pain (3). Furthermore, 20 - 30% of patients experience postoperative nausea and vomiting (PONV) after general anesthesia, often finding it more distressing than pain itself (4).

Traditionally, opioids and nonsteroidal anti-inflammatory drugs (NSAIDs) have been employed to alleviate pain following surgery. However, the high doses required to control pain are associated with numerous adverse effects (5). For example, NSAIDs contribute to 30% of hospital admissions due to bleeding, myocardial infarction, stroke, or renal injury (6). There are also mounting concerns regarding opioid misuse and its health consequences (7, 8). These complications underscore the urgent need for safer alternatives.

Recent guidelines endorse a multimodal approach that combines non-opioid medications, various anesthesia techniques, and nonpharmacological interventions to reduce opioid and NSAID-related side effects and improve pain management (9). For instance, intravenous dexamethasone combined with prophylactic antiemetics such as ondansetron and metoclopramide can effectively reduce PONV and decrease the need for additional antiemetic therapy (10).

The exploration of novel analgesic agents for postoperative pain and PONV includes nicotine, a potent stimulant predominantly found in tobacco plants. Nicotine has demonstrated analgesic properties in both animal models and humans (11). The exact mechanisms underlying nicotine-induced analgesia are not fully understood; however, it is generally believed to involve the activation of nicotinic receptors, particularly the α4β2 subtype, which are distributed throughout the central and peripheral nervous systems and modulate neurotransmitters such as norepinephrine, dopamine, and endogenous opioids (12, 13).

Given this growing body of evidence, it is reasonable to consider nicotine as a potential adjunct for postoperative pain management (14, 15). Nicotine can be administered via transdermal or nasal routes, avoiding interference with the surgical site. Its use may reduce postoperative opioid requirements. Moreover, since nonsmokers are more susceptible to PONV, perioperative nicotine may help decrease its incidence (16). Recent studies assessing nicotine as a postoperative analgesic have yielded inconclusive findings, highlighting the need for further research on its efficacy and safety (13, 17).

## 2. Objectives

This systematic review and narrative synthesis aims to evaluate the effects of perioperative nicotine on postoperative pain and PONV in patients undergoing general anesthesia, potentially contributing to the development of new clinical guidelines and reinforcing evidence-based practice.

## 3. Methods

### 3.1. Protocol and Registration

This systematic review was prospectively registered with PROSPERO (registration number: CRD42024518698).

### 3.2. Data Source

To ensure methodological rigor and transparency, comprehensive searches were conducted in PubMed, Google Scholar, and the Cochrane Central Register of Controlled Trials (CENTRAL) using electronic search engines to identify studies published between 2004 and 2023. The search focused on the effects of perioperative nicotine versus placebo on postoperative outcomes in patients undergoing surgery under general anesthesia.

A synthesis of Medical Subject Headings (MeSH) terms was used, such as: “(Nicotine OR Nicotine Bitartrate OR Nicotine Tartrate) AND (postoperative pain OR perioperative OR analgesia OR post-surgery)”, to capture all relevant studies. Additionally, reference lists of selected studies were reviewed to identify any further eligible studies missed in the initial electronic search.

### 3.3. Study Selection

The PICO framework for this review was as follows:

- Population (P): Patients undergoing general anesthesia

- Intervention (I): Perioperative nicotine administration

- Comparator (C): Placebo or no nicotine administration

- Outcomes (O): The primary outcome was pain scores at various time points. Secondary outcomes included opioid analgesic consumption, PONV incidence, antiemetic requirements, and any other relevant side effects.

Only randomized controlled trials (RCTs) involving patients who underwent surgery under general anesthesia and received nicotine (via intranasal spray or transdermal patch) were included. Exclusion criteria were nonrandomized trials, retrospective and observational studies, abstracts, letters, reviews, studies not involving surgery under general anesthesia, studies not specifically investigating nicotine, and non-English language studies (to avoid methodological inaccuracies and misinterpretation).

### 3.4. Data Extraction

**Figure 1.**
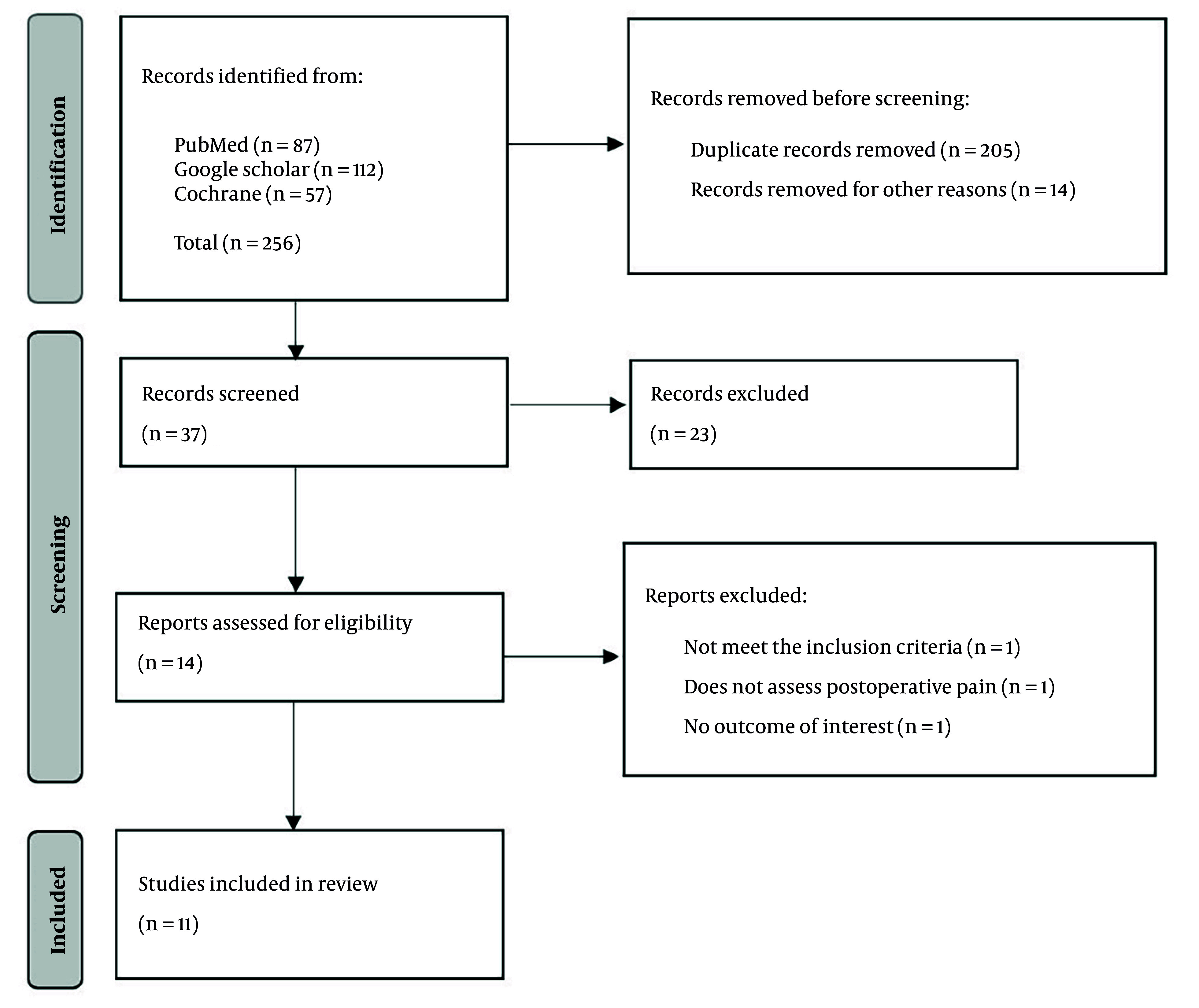
Flow chart for selected studies

As shown in Figure 1, the PRISMA 2020 flow diagram outlines the search and selection process. The initial database search yielded 256 studies: Eighty-seven from PubMed, 112 from Google Scholar, and 57 from the Cochrane Library. After removing 205 duplicates and 15 studies for other reasons, 36 records remained for screening. Fourteen full-text studies were assessed for eligibility after excluding 23 by title and abstract. Two studies were excluded for not assessing postoperative pain or lacking relevant outcomes. Ultimately, eleven studies with 753 patients (384 receiving nicotine, 369 controls) met the inclusion criteria and were included in the final review, followed by narrative synthesis.

All identified studies were uploaded to the Mendeley application to eliminate duplicates. After deduplication, records were imported into Rayyan, where titles and abstracts were independently screened by all authors for relevance. Full-text reviews were then conducted to determine final inclusion. Disagreements were resolved through discussion and consensus to maintain objectivity. Data extracted included author and year, study design, primary and secondary outcomes, sample size, age, gender, smoking status, type of surgery, route and timing of nicotine administration, pain scores and opioid consumption over 24 hours postoperatively, PONV prophylaxis use, and need for rescue antiemetics.

### 3.5. Statistical Analysis and Synthesis

Although a quantitative meta-analysis was initially planned, substantial clinical and statistical heterogeneity precluded meaningful pooled analyses. Therefore, a narrative synthesis was undertaken to address the effectiveness of interventions, the reasons for heterogeneity, and the underlying mechanisms. No subgroup or sensitivity analyses were conducted due to the heterogeneity of interventions and outcomes.

### 3.6. Risk of Bias Assessment

The Cochrane Risk of Bias 2 (RoB 2) tool (18) was used to evaluate the risk of bias in all included studies. Each study was assessed for bias in randomization, intended interventions, missing outcome data, outcome measurement, and selective reporting, with risk classified as low, high, or unclear.

## 4. Results

**Figure 2.**
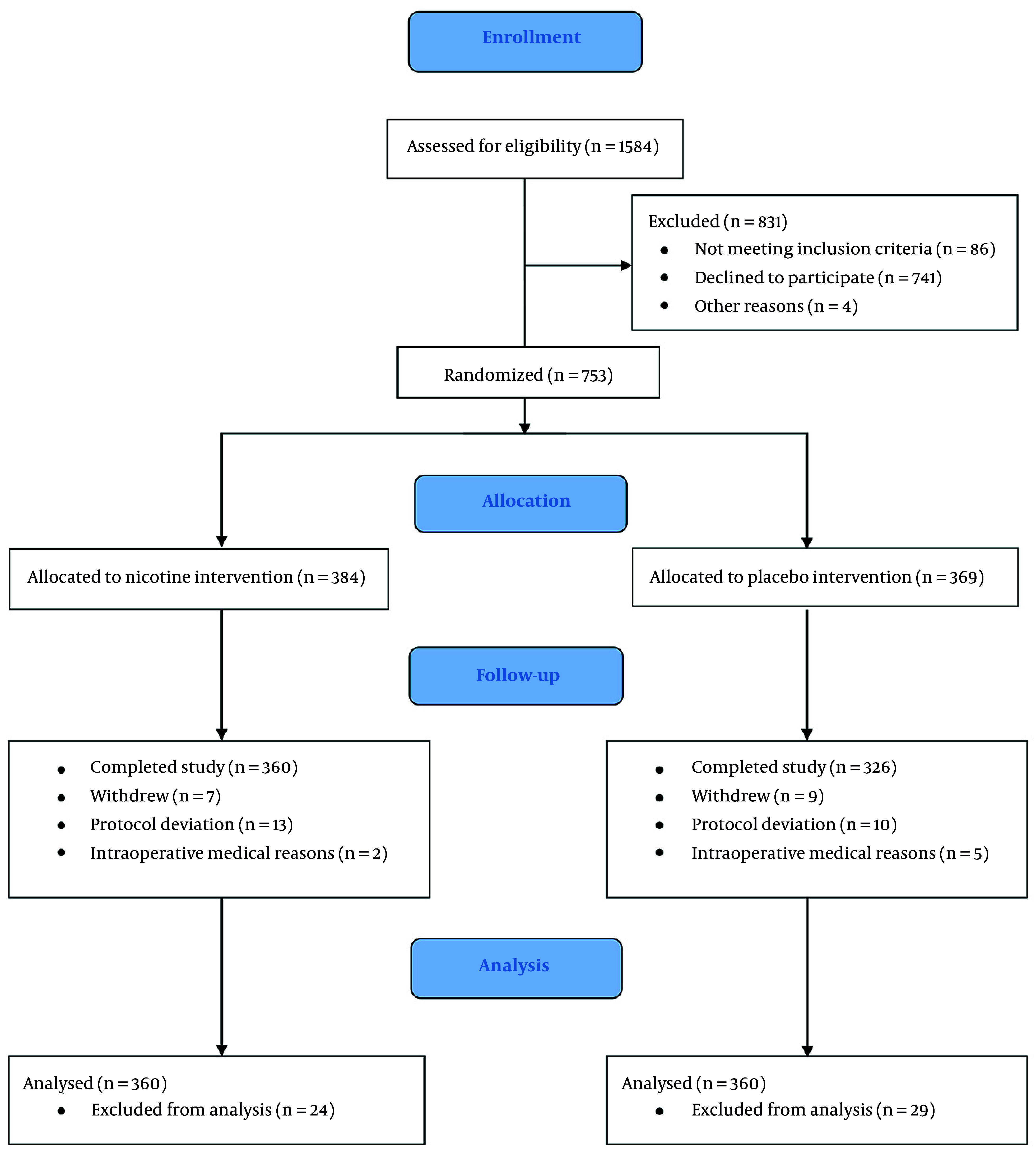
CONSORT diagram of study participants

A total of 1,584 patients were assessed for eligibility from all included RCTs published between 2004 and 2023, encompassing diverse populations (514 female, 239 male) undergoing various surgical procedures. Of these, 686 patients completed the studies and were included in the final analysis (Figure 2). Intranasal sprays and transdermal patches were the most common routes of nicotine administration, with doses ranging from 3 mg (intranasal) to 21 mg (transdermal). The timing of nicotine administration varied, with some studies using preoperative application and others postoperative. Most studies primarily included nonsmokers (n = 629); only a few included smokers, underscoring the need to examine nicotine’s effects across different exposure backgrounds.

Gender distribution was balanced in studies enrolling both sexes, while other trials focused on gender-specific surgeries (for example, gynecological or prostate procedures) and thus included only female or male patients (Table 1). 

**Table 1.** Baseline Features of Included Studies ^a^

Authors, y	Age	Number of Patients	Gender	Smoking Status
**Flood and Daniel, 2004 (** 3 **) **	Placebo: 46 ± 2, nicotine: 43 ± 3	N: 10, C: 10	M: 0, F: 20	S: 0, Ns: 20
**Hong et al., 2008 (** 19 **)**	Placebo: 52 ± 4, nicotine: 48 ± 3	N5: 10, N10: 10, N15: 10, C: 10	M: 20, F: 20	S: 0, Ns: 40
**Turan et al., 2008 (** 20 **)**	Control: 48 ± 13, nicotine: 49 ± 17	N: 43, C: 42	M: 0, F: 85	S: 52, Ns: 33
**Habib et al., 2008 (** 21 **)**	Nicotine: 60 ± 7, placebo: 58 ± 7	N: 44, C: 46	M: 90, F: 0	S: 0, Ns: 90
**Olson et al., 2009 (** 22 **)**	Placebo: 43± 4, nicotine: 46 ± 2	N5: 6, N10: 7, N15: 7, C: 8	M: 6, F: 22	S: 28, Ns: 0
**Czarnetzki et al., 2011 (** 23 **)**	Placebo: 46.8 (15.1), nicotine: 41.8 (13.6)	N: 45, C: 45	M: 53, F: 37	S: 0, Ns: 90
**Jankowski et al., 2011 (** 24 **)**	Placebo: 51 ± 13, nicotine: 50 ± 11	N: 90, C: 89	M: 0, F: 179	S: 0, Ns: 179
**Ibrahim and Dina, 2016 (** 25 **)**	Comparator: 43.6 ± 3.39, nicotine group 1: 143.1 ± 2.4, group 2: 44.3 ± 1.86	N: 40, C: 20	M: 0, F: 60	S: 0, Ns: 60
**Malaithong and Munjupong, 2017 (** 26 **)**	Comparator: 43.86 ± 15.73, nicotine: 45.43 ± 12.98	N: 23, C: 21	M: 24, F: 20	S: 44, Ns: 0
**Martins Filho et al., 2018 (** 27 **)**	Control: 39.75 ± 15.65, nicotine: 31.89 ± 7.17	N: 9, C: 8	M: 3, F: 14	S: 0, Ns: 17
**Seyedsadeghi et al., 2023 (** 28 **)**	Nicotine: 50.74 ± 10.27, placebo: 47.06 ± 11.75	N: 50, C: 50	M: 43, F: 57	S: 0, Ns: 100

Abbreviations: N, nicotine group; C, control group; M/F, male/female; S/NS, smoker/nonsmoker.

^a^ Values are expressed as mean ± SD or No. (%).

The primary outcomes across studies were postoperative pain scores at multiple time points (Table 2). Secondary outcomes included opioid consumption, PONV incidence, and antiemetic use. The most common method of postoperative analgesia was patient-controlled analgesia (PCA) with morphine, although several studies evaluated alternative or adjunctive analgesic methods to reduce opioid-related side effects and promote recovery. While some studies did not specify PONV prophylaxis protocols, most relied on established antiemetics, particularly serotonin receptor antagonists such as ondansetron. A systematic summary of the clinical studies evaluating nicotine’s impact on postoperative outcomes is provided in Table 2. 

**Table 2.** Characteristics and Outcomes of Included Studies ^a^

Authors, y	Study Design	Primary Outcome	Secondary Outcome	Nicotine Route and Dose	PONV Prophylaxis	Postoperative Analgesia
**Flood and Daniel, 2004 (** 3 **)**	A randomized, double blind clinical trial	The patients treated with nicotine reported lower pain scores during the first hour after surgery (peak numerical analog score, (7.6 ± 1.4 versus 5.3 ± 1.6; P < 0.001) and used half the amount of morphine as the control group (12 ± 6 versus 6 ± 5 mg; P < 0.05). Patients who received nicotine still reported less pain than those in the control group 24 h after surgery (1.5 ± 0.5 versus 4.9 ± 1.4; P < 0.01).	Systolic blood pressure was lower in the group that received nicotine (105 ± 3 versus 122 ± 3; P < 0.001), but there was no difference in diastolic blood pressure or heart rate.	Nicotine nasal spray (3 mg), applied before general anesthesia.	Dolasetron (12.5 mg)	PCA morphine
**Hong et al., 2008 (** 19 **)**	A randomized, double-blind, prospective placebo-controlled trial	Patients treated with nicotine reported lower pain scores when compared with those treated with placebo during the first hour after surgery (P = 0.003, average NRS decrease = 1.4, 95% CI = 0.3 - 2.6) and for 5 days after surgery (P = 0.03, average NRS decrease = 1.0, 95% CI = 0.1 - 1.9). There was no increased benefit of nicotine with doses larger than 5 mg. There was a trend suggesting decreased pain medicine use.	NA	Nicotine patch (5, 10, or 15mg/16 h), applied before surgery	Not used	PCA morphine + IV ketorolac for breakthrough pain
**Turan et al., 2008 (** 20 **)**	Randomized clinical trial	Postoperative PCA morphine usage and pain scores while supine or sitting up, intraoperative fentanyl use, oral analgesic consumption, return of bowel sounds, and passage of flatus did not differ between the two groups.	Although ambulation and hospitalization times, as well as quality of recovery scores, did not differ, resumption of oral intake was delayed in the nicotine group. Discharge eligibility scores were higher in the nicotine group at 48 and 72 h compared with the control group, but the time to return to work was 19 days in both treatment groups.	Nicotine patch (5, 10, or 15mg/16 h), applied before surgery	Not used	PCA morphine, then, after 72 hours acetaminophen (500 mg po), in combination with codeine (30 mg po every 6 - 8 h, when needed)
**Habib et al., 2008 (** 21 **)**	A prospective, double-blind, placebo-controlled study	The nicotine group showed significantly lower cumulative morphine consumption at 24 h: 33.3 ± 30.8 mg vs. 44.7 ± 26.4 mg (P = 0.0059, time × treatment P = 0.0031). However, the repeated measures tests found no difference in amount of pain reported on coughing or at rest, either as treatment effects or in interaction with time. In post-hoc comparisons, there was no significant difference in amount of pain reported on coughing or at rest at any of the times assessed.	There were also no significant differences between the groups in the incidence of PONV or the need for rescue antiemetics.	Nicotine patch (21 mg), applied before anesthesia and reapplied at on the second and third postoperative days	Not used	PCA morphine + IV ketorolac (15 mg every 6 h)
**Olson et al., 2009 (** 22 **)**	Randomized, double-blind, prospective, placebo-controlled trial	Patients treated with nicotine reported higher pain scores than those treated with placebo over the first hour after surgery (P < 0.01, Average Numerical Rating Scale increase = 0.67) and there was no difference between groups in the subsequent 5 days (P > 0.05). There was no significant dose effect. Diastolic blood pressure in the first hour was higher in the placebo group compared with the nicotine-treated group (P < 0.01, average increase = 11 mm Hg). There was no difference in nausea or sedation.	NA	Nicotine patch (7 mg), applied before surgery	Ondansetron (4 mg) given within 30 min of the end of surgery	PCA: Morphine or an equivalent dose of hydromorphone or meperidine when needed + ketorolac for breakthrough pain
**Czarnetzki et al., 2011 (** 23 **)**	Randomized, placebo-controlled trial	NA	The incidence of nausea was 22.2% with nicotine and 24.4% with placebo (P = 0.80), and the incidence of vomiting was 20.0% with nicotine and 17.8% with placebo (P = 0.78). Cumulative 24 h incidence of nausea was 42.2% with nicotine and 40.0% with placebo (P = 0.83), and of vomiting was 31.1% with nicotine and 28.9% with placebo (P = 0.81). The PONV episodes tended to occur earlier in the nicotine group. Postoperative headache occurred in 17.8% of patients treated with nicotine and in 15.6% with placebo (P = 0.49). More patients receiving nicotine reported a low quality of sleep during the first postoperative night (26.7% vs. 6.8% with placebo; P = 0.01).	Transdermal nicotine patch (5, 10, or 15 mg/16 h), applied for 24 h	Ondansetron (4 mg) during general anesthesia	Morphine, paracetamol, and ketorolac or ibuprofen.
**Jankowski et al., 2011 (** 24 **)**	A double-blind, randomized placebo-controlled trial	Opioid requirements did not differ between the nicotine and placebo groups for either inpatients or outpatients. In patients who received nicotine were more likely to receive antiemetic rescue medications (P = 0.009) and report higher NVDS scores (P = 0.025).	In patients who received intranasal nicotine used less opioid. From an overall analysis, patients in the nicotine group were more likely to experience nausea (71.1 vs. 56.2% P = 0.044), receive rescue antiemetics (57.8 vs. 38.2% P = 0.011), and report higher nausea verbal descriptive scores [2 (0, 2); vs. 1 (0, 2), P = 0.006] in PACU. In patients who received nicotine were more likely to receive antiemetics (P = 0.009).	Transcutaneous nicotine (7 mg), applied 1 h before surgery and left in place for 24 h	Not used	PCA: Morphine or fentanyl and then oxycodone or hydrocodone. Also, paracetamol (1 g, orally every 6 h) and ketorolac (15 mg), both as PRN
**Ibrahim and Dina, 2016 (** 25 **)**	A randomized controlled double-blind	There was a significant reduction in the VAS score, total pethidine requirements (mg) and significantly higher patient’s satisfaction in TDN and TDM groups when compared with the C group postoperatively.	The sedation score and surgeons’ satisfaction were significantly higher associated with a significant decrease in MAP and Intraoperative bleeding in TDM group compared to C and TDN groups postoperatively. Significant nausea and vomiting in TDN group and significant sedation in TDM group were recorded.	Intranasal nicotine spray (3 mg), applied immediately after the end of surgery but before emergence from anesthesia	Not used	PCA pethidine
**Malaithong and Munjupong, 2017 (** 26 **)**	A prospective, double-blind, placebo-controlled study	There was no significant difference in mean NRS and average opioid consumption at 1 hour and 24 hours postoperatively between controlled and treatment group. However, the significant reduction in average NRS from baseline at 1 hour and 24 hours postoperatively were found in both groups (P < 0.001).	NA	Transdermal nicotine patch (15 mg) and melatonin patch (7 mg), applied 2 h before surgery and removed after 12 h	Granisetron (1 mg IV)	PCA morphine
**Martins Filho et al., 2018 (** 27 **)**	An analytical, prospective, randomized, triple-blinded, clinical study	Regarding the pain parameter, there was no statistically significant difference between the groups (P > 0.05).	Taking into account the nausea parameter, there was no statistically significant difference between the groups (P > 0.05). Also, the evaluation of rescue medication, both opioids and prokinetics, did not show any significant statistical difference between the groups. Among the hemodynamic parameters, there was only one statistically significant difference in the analysis of oxygen saturation and systolic blood pressure (SBP) six hours after surgery: The mean oxygen saturation was higher in the test group (97.89 × 95.88) and the mean SBP was higher in the control group (123.89 × 110.0).	Transdermal patch (17.5 mg, with 7 mg nicotine in 24 h), applied before induction of anesthesia	Ondansetron (8 mg)	PCA valdecoxib + paracetamol (750 mg orally every 6 h) + morphine (0.1mg/kg) as PRN
**Seyedsadeghi et al., 2023 (** 28 **)**	Triple-blind clinical trial.	There was also no statistically significant difference between the two groups in terms of analgesics (P = 0.096).	There was also no statistically significant difference between the two groups in terms of antiemetics (P = 0.1). Moreover, the frequency of severe nausea and vomiting during the study in the nicotine group was higher than in the placebo group (4 vs. 1) but this difference was not statistically significant (P > 0.05).	Nicotine patch (14 mg)	Ondansetron	PCA morphine

Abbreviations: N, nicotine group; N5, N10, and N15, groups taking nicotine patches at 5, 10, and 15 mg/16 h, respectively; C, control group; M/F, male/female; S/NS, smoker/nonsmoker; PONV, postoperative nausea and/or vomiting; PCA, patient-controlled analgesia; PRN, when necessary; VAS, Visual Analog Scale; NRS, Numeric Rating Scale.

^a^ Values are expressed as mean ± SD.

All studies evaluated using the RoB 2 tool were found to have a low risk of bias across all domains (including randomization, intervention, missing data, outcome assessment, and selective reporting), as detailed in Table 3 (3, 19-28).

**Table 3.** Risk of Bias Assessment for the Included Studies

References, y	Randomization Process and Allocation Concealed	Blinding of Participants and Investigators	Blinding of Outcome Assessment	Missing Outcome Data	Selecting Reporting
**Flood and Daniel, 2004 (** 3 **)**	Low	Low	Low	Low	Low
**Hong et al., 2008 (** 19 **)**	Low	Low	Low	Low	Low
**Turan et al., 2008 (** 20 **)**	Low	Low	Low	Low	Low
**Habib et al., 2008 (** 21 **)**	Low	Low	Low	Low	Low
**Olson et al., 2009 (** 22 **)**	Low	Low	Low	Low	Low
**Czarnetzki et al., 2011 (** 23 **)**	Low	Low	Low	Low	Low
**Jankowski et al., 2011 (** 24 **)**	Low	Low	Low	Low	Low
**Ibrahim and Dina, 2016 (** 25 **)**	Low	Low	Low	Low	Low
**Malaithong and Munjupong, 2017 (** 26 **)**	Low	Low	Low	Low	Low
**Martins Filho et al., 2018 (** 27 **)**	Low	Low	Low	Low	Low
**Seyedsadeghi et al., 2023 (** 28 **)**	Low	Low	Low	Low	Low

## 5. Discussion

The main objective of this systematic review was to map and synthesize current evidence on perioperative nicotine administration and its effects on postoperative pain management and PONV in patients undergoing general anesthesia. Animal studies have indicated that nicotine possesses antinociceptive properties (29). Our findings for the primary outcome indicate mixed evidence regarding the impact of nicotine on postoperative pain, nausea, and vomiting. This complexity reflects the challenges associated with utilizing nicotine as an analgesic.

For instance, several studies — Flood and Daniel (3), Ibrahim and Dina (25), Jankowski et al. (24), and Hong et al. (19) — reported significant reductions in pain levels, as measured by Numeric Rating scales (NRS). Conversely, other studies — Malaithong and Munjupong (26), Olson et al. (22), Turan et al. (20), and Seyedsadeghi et al. (28) — found no significant reduction in postoperative pain. Martins reported a decrease in pain at 24 hours, but this was not statistically significant (27). These discrepancies may stem from variations in nicotine dosage, timing of administration, plasma nicotine levels, patient demographics, and smoking status.

The opioid-sparing effect of nicotine was also examined, with differing outcomes. Flood and Jankowski found that intranasal nicotine significantly reduced opioid consumption (3, 24). Transdermal nicotine patches provide more consistent plasma levels compared to intranasal sprays, which may contribute to improved pain management (22). However, other studies reported no significant reduction in opioid consumption with transdermal nicotine, even when postoperative pain was reduced (20, 22, 26). This suggests that the route of administration may be a key factor, likely due to differences in pharmacokinetics and pharmacodynamics.

Previous research has consistently linked nicotine with an increased risk of PONV, particularly among nonsmokers (29-31), a finding supported by Jankowski and Czarnetzki (23, 24). Some studies suggested a dose-response relationship; one found that doses exceeding 5 mg were associated with increased nausea, although not statistically significant (19). Both intranasal and transdermal nicotine delivery can induce nausea, but transdermal administration may carry a higher risk because it maintains elevated plasma nicotine concentrations for longer periods (21).

Despite potential analgesic effects, nicotine commonly causes PONV, often necessitating antiemetic use. Several studies observed increased use of antiemetics such as ondansetron, dolasetron, and granisetron (3, 21, 22, 25, 27). Therefore, comprehensive antiemetic protocols may be required when nicotine is used perioperatively as part of a multimodal pain regimen.

This review has several strengths, including the inclusion of diverse patient populations and surgical procedures. Nonetheless, there are notable limitations. The use of perioperative corticosteroids was inconsistently reported and not standardized, introducing a confounding factor given their analgesic and antiemetic effects. Additionally, the lack of plasma nicotine concentration measurements and direct comparisons between dosage forms (patch versus spray) in some studies limits the ability to accurately assess safety, efficacy, and adverse effects. The variability in routes and dosages further limited the strength and interpretability of the narrative synthesis and precluded formal meta-analysis.

Another limitation is the focus on female patients and the exclusion of smokers in many studies. Given known differences in pain perception, nicotine metabolism, and side effect incidence between genders and smoking statuses, this omission restricts the generalizability of findings. Future longitudinal studies should aim to standardize nicotine dosages, routes of administration, and PONV prophylaxis protocols, and include a broader range of patient demographics. Additionally, research should address the long-term effects of perioperative nicotine use on recovery and rehabilitation outcomes.

### 5.1. Conclusions

There is growing evidence that perioperative nicotine administration may significantly reduce postoperative pain, analgesic consumption, nausea, and vomiting in patients undergoing surgery under general anesthesia. However, these findings should be interpreted with caution, as some studies have reported contrary effects. Future clinical trials employing rigorous, standardized methodologies are needed to improve evidence-based practice and optimize postoperative care.

## Data Availability

The dataset presented in this study is available on request from the corresponding author during submission or after publication. The data are not publicly available due to privacy concerns.
